# Impact of age on the reliability of GE Entropy™ module indices for guidance of maintenance of anaesthesia in adult patients: a single-centre retrospective analysis

**DOI:** 10.1016/j.bja.2024.11.050

**Published:** 2025-02-06

**Authors:** Max Ebensperger, Matthias Kreuzer, Stephan Kratzer, Gerhard Schneider, Stefan Schwerin

**Affiliations:** 1 Department of Anesthesiology and Intensive Care, TUM School of Medicine and Health, Technical University of Munich, Munich, Germany; 2 Department of Anesthesia and Intensive Care Medicine, Hessing Foundation, Augsburg, Germany

**Keywords:** burst suppression, electroencephalography, neuromonitoring, response entropy, state entropy

## Abstract

**Background:**

The GE Entropy™ module uses frontal EEG to compute the processed indices state entropy (SE), response entropy (RE), and burst suppression ratio (BSR) to guide maintenance of anaesthesia by supposedly minimising overly ‘*deep*’ or ‘*light*’ anaesthesia. It remains unclear whether the manufacturer-recommended index ranges accurately reflect anaesthesia levels or prevent complications such as burst suppression or arousal reactions.

**Methods:**

We retrospectively analysed 15 608 patient records, evaluating 14 770 adult patients (18–90 yr old) undergoing general anaesthesia. Age-dependent effects on processed index values were assessed using linear regression and Spearman's correlation coefficients (rho).

**Results:**

During steady-state anaesthesia (BSR=0), only 38.4% (32.5–42.4%) of SE values were within the recommended range, with most values below the target. Age was positively associated with an increase in age-adjusted minimal alveolar concentration for volatile anaesthetics (adjusted [adj.] *R*^2^=0.18, *P*<0.001, rho=0.47 [0.20–0.70]). Despite this, SE paradoxically increased with age (adj. *R*^2^=0.45, *P*<0.001, rho=0.67 [0.51–0.79]). This trend persisted even during periods with positive BSR despite supposedly adequate SE values (adj. *R*^2^=0.73, *P*<0.001, rho=0.90 [0.80–0.95]). Maintaining anaesthesia within the recommended index range did not prevent positive BSR. Additionally, both frequency (adj. *R*^2^=0.70, *P*<0.001, rho=0.92 [0.85–0.95]) and duration (adj. *R*^2^=0.73, *P*<0.001, rho=0.89 [0.82–0.93]) of ΔRE-SE≥10, indicating arousal, increased with age.

**Conclusions:**

Despite their intuitive appeal, the processed EEG index values SE, RE, ΔRE-SE, and BSR showed limited reliability in guiding maintenance of anaesthesia, especially in older patients. Anaesthesiologists should not rely exclusively on the recommended index value range, as it is often unattainable and does not prevent burst suppression or arousal indicators.

**Editor's key points**

• Processed EEG neuromonitoring indices are used to help guide anaesthetic dosing to avoid inadequate or excessive levels of anaesthesia, both of which can be detrimental.
• In this single-centre retrospective analysis, the authors analysed processed EEG indices from 14 770 adult patients undergoing general anaesthesia.
• There were significant age-dependent effects on processed index values. Adherence to the recommended adequate index range did not prevent burst suppression, and evidence of arousal occurred even in the adequate anaesthesia range, especially in older patients.
• Relying solely on these processed EEG indices poses a risk of both underdosing and overdosing during maintenance of anaesthesia, particularly in older patients.

Neuromonitoring during general anaesthesia has become standard practice in many countries and is recommended by numerous medical associations.^1^^,^^2^ However, the benefit of commercial systems is controversial.^3^ These systems capture frontal EEG signals from the forehead and compute a processed index, which is inversely correlated to the reductive ‘*depth*’ of anaesthesia concept (high index–low ‘*depth*’ of anaesthesia, low index–high ‘*depth*’ of anaesthesia).^4^^,^^5^ The GE Entropy™ module (GE Healthcare, Helsinki, Finland) presents two indices: response entropy (RE) and state entropy (SE). Although SE and RE use spectral entropy, they focus on different frequency ranges based on different time intervals. RE is calculated to encompass high frequencies (0.8–47 Hz) influenced by facial EMG, while SE (0.8–32 Hz) captures the hypnotic component of anaesthesia. RE is normalised on a range from 0 to 100, whereas SE spans from 0 to 91.^5^ The manufacturer defines the SE range for adequate anaesthesia during steady-state conditions as BSR=0, SE=40–60,^6^ which is widely adopted in research and clinical practice.^7^^,^^8^ However, the validity of these predefined limits has been progressively called into question, as both low bispectral index (BIS) and SE/RE values have falsely indicated adequate anaesthesia despite wakefulness in healthy volunteers under neuromuscular block.^9^^,^^10^ In a prospective study, titrating anaesthesia to match the recommended SE range proved unsuccessful.^11^ Further, the comparability of different commercial indices seems limited.^12^, ^13^, ^14^

EEG neuromonitoring indices are supposed to help avoid inadequate levels of anaesthesia. The systems display the burst suppression ratio (BSR) to signal excessively ‘*deep*’ anaesthesia. The RE-SE difference (ΔRE-SE) might contain valuable information regarding ‘*light*’ levels of anaesthesia.^5^ ΔRE-SE ≥10 is hypothesised to indicate arousal. However, this cut-off value is currently not well validated, and it is unclear whether changes are primarily a result of insufficient anaesthesia, analgesia, or neuromuscular block.^15^, ^16^, ^17^ As a major limitation, processed EEG indices neither incorporate patient characteristics nor the anaesthesia regimen applied. This is highly problematic, as there is substantial evidence that older patients tend to have increased power in faster frequency bands. This effect artificially mimics the EEG of a less anaesthetised brain,^18^ ultimately leading to higher processed EEG index values.^19^^,^^20^ The processed EEG indices provided by the GE Entropy^TM^ module (SE, RE, and BSR) might have limited reliability for guiding anaesthesia *‘depth’*, especially in older patients. The manufacturer-recommended index ranges might not accurately reflect adequate anaesthesia levels or prevent complications such as burst suppression or arousal reactions, highlighting a need for careful interpretation and a multimodal approach.

We used a large dataset of processed intraoperative EEG to verify how reliably the indices RE, SE, ΔRE-SE≥10, and BSR perform with respect to the underlying premise that prefrontally-derived EEG can effectively delineate the spectrum from adequate to overly ‘*deep*’ or ‘*light*’ anaesthesia, irrespective of patient characteristics and anaesthesia regime applied. We further examined age-dependent changes of these indices and analysed cases with contradictory outputs, such as constellations where SE suggested adequate anaesthesia despite simultaneous BSR ≥5 or ΔRE-SE ≥10 during maintenance anaesthesia.

## Methods

### Ethics approval

The institutional ethics committee of the Technical University of Munich/Klinikum rechts der Isar approved the study on December 12, 2018 (No. 572/18s) and waived the written informed consent requirement. Electronic records from 2016 to 2017 were included for analysis. This manuscript adheres to applicable Strengthening the Reporting of Observational Studies in Epidemiology (STROBE) guidelines.^21^

### Electronic records

In this retrospective investigation, we screened processed intraoperative EEG data derived from a total of 15 608 patients who underwent general anaesthesia at the Technical University of Munich Hospital (Munich, Germany). 14 770 patient records fulfilled our inclusion criteria (Supplementary Fig. S1). The institution's electronic records contained RE, SE, and BSR values from the GE Entropy™ module. Dosages of volatile anaesthetics (sevoflurane and desflurane) were stored as end-expiratory volume percentages (${eeVol}_{\left(\%\right)}$). We used an adapted version of the formula described by Nickalls and Mapleson^22^ to calculate the corresponding age-adjusted minimal alveolar concentration (aaMAC):

aaMAC=eeVol(%)MAC(40)×10(−0.00269×(age−40))

Quadruplets of RE, SE, BSR, and, if applicable, corresponding aaMAC values were concatenated at 10-s intervals. The records further contained patient characteristics and anaesthesia-related information, including age, sex (male or female), whether total i.v. anaesthesia (TIVA) or volatile anaesthesia were used for anaesthesia maintenance, and overall surgery duration. The respective perioperative time stamps, including the start of anaesthesia, end of anaesthesia induction, surgical incision, and end of anaesthesia, follow the specifications of the German Perioperative Procedural Time Glossary.^23^

### Processed EEG thresholds

In the absence of EMG activation, RE and SE should coincide. A difference of >10 in ΔRE-SE has been suggested as a threshold for arousal, necessitating anaesthesia dosing.^24^^,^^25^ BSR is calculated independently of the entropy indices using a nonlinear energy operator (NLEO) derived from computing two EEG frequency bands within segments lasting 0.05 s. When NLEO remains below a specific threshold, the BSR (0–100) is presented.^26^ Though there is no universally accepted convention, suppression periods as short as 1 s were used for identification of burst suppression.^27^ Consistent with previous studies,^28^ we used BSR ≥5 as a threshold, indicating 3 s of suppressed EEG within 1 min.

### Perioperative time intervals

We defined three distinct perioperative time intervals for subsequent analyses. The ‘*surgical period*’ exclusively includes data from time points during anaesthesia maintenance ranging from 5 min before ‘*surgical incision*’ to 10 min before ‘*end of anaesthesia*’. We hereby excluded potentially transient states found to distort the data during our initial analyses. The second interval, labelled ‘*steady-state anaesthesia*’, a subgroup of the ‘*surgical period*’, only includes simultaneous SE and RE indices <80 with a corresponding BSR=0. For our analysis of aaMAC values, the ‘*surgical period*’ and subsequent ‘*steady-state anaesthesia*’ were more narrowly defined by including only values recorded from 40 min after the start to 20 min before the end of anaesthesia. This adjustment further compensated for the onset and offset kinetics of volatile anaesthetics. For the third interval (‘*burst suppression*’), we examined SE and RE values with a simultaneous BSR ≥5 during the ‘*surgical period*’.

### Statistical analysis

Continuous data are presented as medians with first and third quartiles (Q1, Q3), while categorical data are shown as absolute numbers with percentages. We performed linear regression analyses using MATLAB's ‘*fitlm*’ function to evaluate the association between age and SE/RE values, including 95% confidence intervals (CI) for slope coefficients. In cases where low *R*^2^ values indicated a suboptimal linear fit, we fit second-degree (*‘poly2’*) and third-degree polynomial models (*‘poly3’*). We then performed a likelihood ratio test between the models (MATLAB function *‘compare’*), which included Akaike information criterion (AIC) and Bayesian information criterion (BIC) values. This allowed us to evaluate whether the higher-degree polynomials significantly improved the fit.

Significance level was set at *P*<0.05. Spearman's rank correlation coefficients (rho) with corresponding CI were determined using 10 k-fold bootstrapping. Additionally, we computed adjusted *R*^2^ values. To determine the discriminatory ability of SE values with respect to burst suppression occurrence for the surgical period, BSR values were first dichotomised (BSR >0 or BSR=0) and then grouped with respective SE values. We calculated the area under the receiving operating characteristic (AUROC) metrics using MATLAB's ‘*perfcurve*’ function. All analyses and visualisations were performed using MATLAB R2023a (Mathworks, Natick, MA, USA).

### Missing data and bias

To avoid selection bias, we included all data sets that exhibited complete records of all concurrent metric combinations (SE, RE, BSR, and, if applicable, ${eeVol}_{\left(\%\right)}$). Patients were excluded if they exhibited only invalid (non-numerical or missing) entries for one or more specified metrics (Supplementary Fig. S1). We also excluded data sets of procedures >8 h and patients >90 yr because of the small sample size and the high percentage of incomplete data for both categories (Supplementary Fig. S2). All remaining invalid index values, such as non-numerical entries, were excluded from arithmetic functions, statistical analyses, and plots.

## Results

### Patient characteristics

Our analysis included patients from 18 to 90 yr of age; 54% were male and 46% were female. The median patient age was 59 (43–72) yr. Median surgery duration was 120 (79–179) min. Median BMI was 25.6 (22.8–29) kg m^−2^, and the American Society of Anesthesiologists (ASA) physical status distribution was: ASA 1: 23%, ASA 2: 49%, ASA 3: 26%, ASA 4: 1.92%, ASA 5: 0.08%

### Age-dependent increases in mean state entropy and response entropy values during steady-state anaesthesia

**Fig 1:**
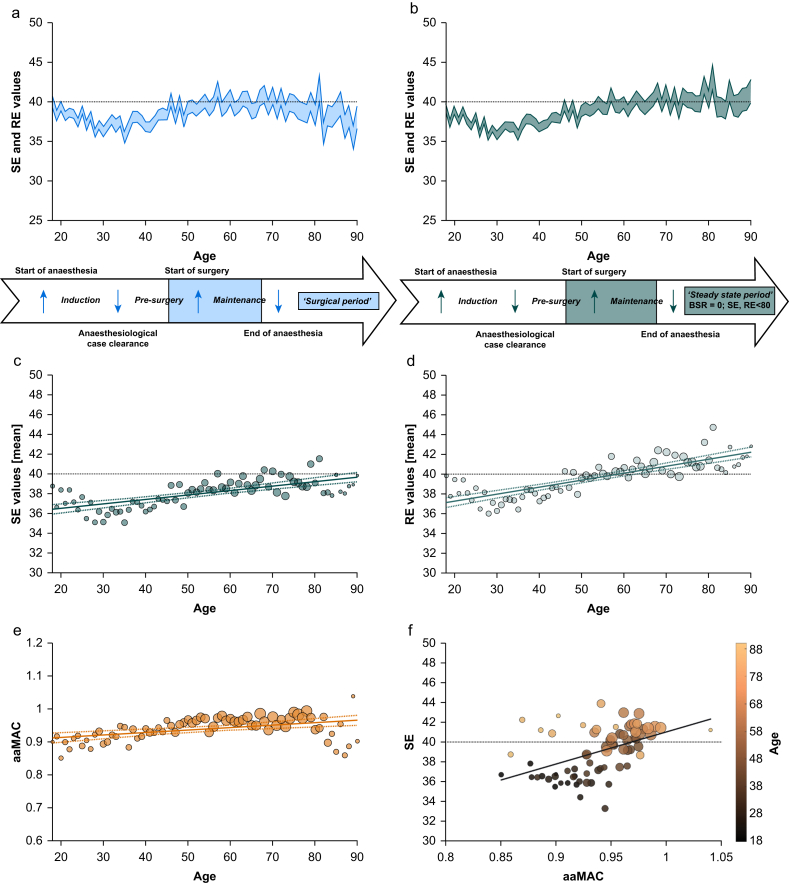
State entropy (SE) and response entropy (RE) for the surgical and steady-state anaesthesia period with corresponding volatile anaesthetic concentrations. The lower threshold for adequate anaesthesia, as recommended by the manufacturer (40), is indicated with a dotted line. Dot sizes per age are scaled according to group size. (a) Mean SE (dark blue) and RE (light blue). The ΔRE-SE corresponds to the shaded area. Below, a timeline indicates the respective time period. (b) Mean SE (dark green) and RE (light green). (c) Mean SE values as a function of age. Linear regression fit with corresponding confidence intervals as dotted lines. (d) Mean RE values as a function of age. (e) Mean age-adjusted minimal alveolar concentration (aaMAC) values for sevoflurane as a function of patient age for the steady-state period. (f) Mean SE values at mean aaMAC grouped per patient age for the steady-state period. BSR, burst suppression ratio.

RE and SE values increased with age for both the surgical period (Fig. 1a) and during steady-state anaesthesia (Fig. 1b). This resulted in a baseline increase of 0.13% for SE and 0.20% for RE per year of age (Fig. 1c and d). Linear regression analyses showed significant increases with age for both indices (SE: rho=0.67 [0.51–0.79], adj. *R*^2^=0.45, *P*<0.001; RE: rho=0.84 [0.76–0.89], adj. *R*^2^=0.66, *P*<0.001). See Table 1 for all linear regression models and corresponding correlation coefficients. Because of the relatively low adjusted *R*^2^ values observed in the linear models, we proceeded to fit second-degree and third-degree polynomial models to test whether the relationships between SE and age, and RE and age, were better described by nonlinear functions. Likelihood ratio tests indicated that the third-degree polynomial model significantly improved both fits (see Supplementary Tables S1 and S2 and Supplementary Fig. S3). Mean SE values, supposed to reflect the hypnotic component of anaesthesia, persistently remained below the manufacturer-defined threshold for adequate anaesthesia of 40^6^ during steady-state anaesthesia (BSR=0). The age-dependent increase in mean SE values only partially recouped this trend.

**Table 1:** Results of linear regression models with corresponding Spearman's rank correlation coefficients (rho). All linear models describe the relationships of parameter constellations/combinations detailed in the first column and patient age. Results below the double line correspond to linear regression analyses detailed in Supplementary Figures. aaMAC, age-adjusted minimal alveolar concentration; adj. *R*^2^, adjusted *R*^2^; BSR, burst suppression ratio; CI, confidence interval; RE, response entropy; SE, state entropy; TIVA, total i.v. anaesthesia.

	Linear model (95% CI of y-intercept)	Coefficient slope 95% CI	Adj. *R*^2^	*P*-values	rho (95% CI)
RE (steady-state)	0.071×age+35.85	0.059–0.083	0.66	<0.001	0.84 (0.76–0.89)
SE (steady-state)	0.045×age+35.63	0.034–0.057	0.45	<0.001	0.67 (0.51–0.79)
aaMAC (steady-state)	0.0007×age+0.90	0.0004–0.0011	0.18	<0.001	0.47 (0.20–0.70)
SE over aaMAC (steady-state)	32.332×aaMAC+8.70	18.402–46.261	0.22	<0.001	0.54 (0.33–0.71)

% 60<SE<80	0.022×age+3.18	−0.006 to 0.051	0.02	0.13	0.03 (−0.006 to 0.05)
% 40≤SE≤60	0.092×age+32.65	−0.002 to 0.187	0.04	0.05	0.38 (0.11–0.62)
% 0 ≤SE<40	−0.225×age+68.76	−0.337 to −0.114	0.18	<0.001	−0.46 (−0.68 to −0.20)

% 40≤SE≤60 at BSR ≥5	0.059×age-1.36	0.051–0.068	0.73	<0.001	0.90 (0.80–0.95)
Consecutive s 40≤SE≤60, BSR ≥5	0.610×age-9.90	0.492–0.728	0.60	<0.001	0.84 (0.73–0.91)
Cumulative min 40≤SE≤60, BSR ≥5	0.040×age-1.24	0.033–0.046	0.70	<0.001	0.95 (0.91–0.97)

RE-SE (steady state)	0.026×age+0.22	0.023–0.028	0.85	<0.001	0.96 (0.93–0.97)
% ΔRE-SE ≥10	0.062×age-0.39	0.052–0.071	0.70	<0.001	0.92 (0.85–0.95)
Consecutive s ΔRE-SE≥10	0.466×age+25.60	0.303–0.629	0.30	<0.001	0.59 (0.39–0.73)
Cumulative min ΔRE-SE≥10	0.041×age+0.02	0.035–0.047	0.73	<0.001	0.89 (0.82–0.93)

RE-SE (Gas)	0.030×age+0.20 (0.02–0.39)	0.027–0.034	0.83	< 0.001	0.93 (0.86–0.96)
RE-SE (TIVA)	0.027×age+0.80 (0.65–094)	0.024–0.029	0.87	< 0.001	0.94 (0.89–0.96)

40≤SE≤60 (steady-state)	0.018×age+45.65	0.011–0.025	0.29	<0.001	0.52 (0.30–0.67)

To address the objection that the observed increase in the indices in older patients might be attributable to lower anaesthetic concentrations, we analysed their relationship with mean aaMAC values. We actually found a moderately positive correlation between aaMAC values and patient age during steady-state anaesthesia (rho=0.47 [0.20–0.70], adj. *R*^2^=0.18, *P*<0.001, see Fig. 1e). This suggests that intrinsic, age-related changes in EEG are the primary reason for higher SE values. Additionally, we confirmed that patients were not systematically overdosed with volatile anaesthetics, as the mean aaMAC values for sevoflurane remained consistently below 1.0 across all age groups. Despite receiving a higher mean aaMAC, older patients displayed higher mean SE values, see Fig. 1f. For the age-dependent relationship between mean aaMAC and mean SE during steady-state anaesthesia, see Supplementary Figure S4.

### Alignment of state entropy values with the manufacturer-recommended index range for adequate anaesthesia

**Fig 2:**
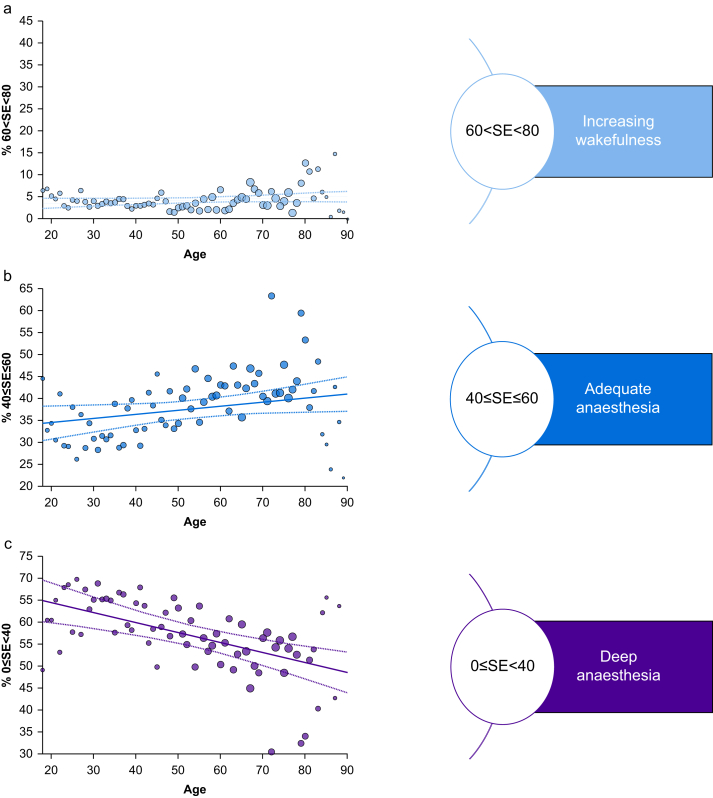
Percentage distribution of state entropy (SE) values during steady-state anaesthesia. The scale on the right was modified according to the GE Entropy™ description. Linear regression fits in corresponding colours with confidence intervals as dotted lines. Dot sizes per age are scaled according to group sizes. (a) Percentage of SE values between 60 and 80, corresponding to an increasing chance of potential wakefulness and recall. (b) Percentage of SE values between 40 and 60 at BSR=0, deemed adequate for most surgical procedures. (c) Percentage of SE values between 0 and 40 at BSR=0, tagged as ‘*deep anaesthesia*’.

Only 38.4% (32.5–42.4%) of all recorded SE values during steady-state anaesthesia (BSR=0) fell within the manufacturer-recommended range for ‘*adequate*’ anaesthesia (Fig. 2b). SE values ranging from 0 to 40 accounted for 57.5% (52.9–63.7%) of all observations (Fig. 2c), while SE values ranging from 60 to 80 were observed in only 4% (2.9–5.1%) (Fig. 2a). Supplementary Figure S5 illustrates the distribution of mean SE values within the relative ranges. Following the manufacturer's recommendations, most SE index values recorded during steady-state anaesthesia must be considered too low. The age-dependent increase in SE values partially recouped this effect.

### Titrating anaesthesia to the manufacturer-recommended range does not prevent concurrent burst suppression

**Fig 3:**
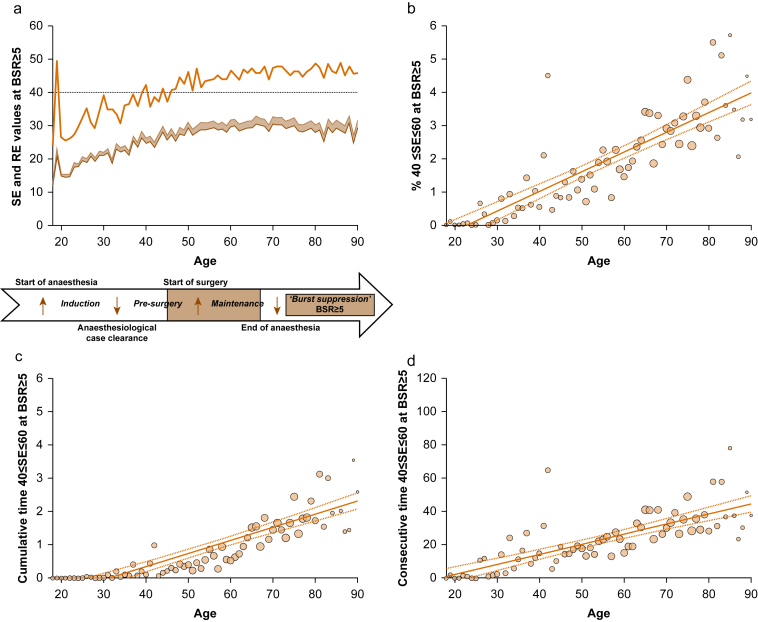
State entropy (SE) and response entropy (RE) with simultaneous burst suppression ratio (BSR) ≥5 as a function of age. (a) Mean SE (dark yellow) and RE (light yellow) values with simultaneous BSR≥5. ΔRE-SE as a shaded area. The 95^th^ percentile of mean SE values (brown). A dotted line shows the lower threshold for adequate anaesthesia (SE 40). Below, a timeline delineates the surgical period. (b) Mean percentage of SE values within the 40–60 range despite BSR ≥5. (c) Cumulative time (min) of SE values 40–60 with simultaneous BSR ≥5. (d) Consecutive time (s) of SE values 40–60 with simultaneous BSR ≥5.

To evaluate monitor reliability in helping to guide anaesthesia to safe levels, we examined the behaviour of SE and RE values with simultaneously recorded BSR ≥5 as a function of age (Fig. 3a). One would expect that during burst suppression, SE values should always be low. In patients aged 20–40 yr, mean SE values ranged between 15 and 25. Intriguingly, mean SE index values increased with age and plateaued at around SE=30. When incorporating the mean SE value 95^th^ percentiles during BSR ≥5, contradictory monitor outputs emerged (i.e. the display of ‘*adequate*’ anaesthesia [SE=40–60] together with BSR ≥5 in patients aged 50 yr and above). We observed a significant increase in the percentage of contradictory value combinations and a very strong positive correlation with patient age (age *vs* SE=40–60 at BSR ≥5: rho=0.90 [0.80–0.95], adj. *R*^2^=0.73, *P*<0.001, Fig. 3b). Older patients spent more cumulative and consecutive time in these contradictory combinations (Fig. 3c and d). Supplementary Figure S6a and b show the distribution of all recorded BSR values with corresponding SE and RE values as a function of age. The AUC=0.76 indicated only a fair effect of SE to discriminate situations of BSR=0 from BSR >0 (Supplementary Fig. S6c). Even when titrating anaesthesia to the manufacturer-recommended SE index range, simultaneous BSR ≥5 occurred, particularly in older patients.

### Titrating anaesthesia to the manufacturer-recommended range does not prevent concurrent arousal

**Fig 4:**
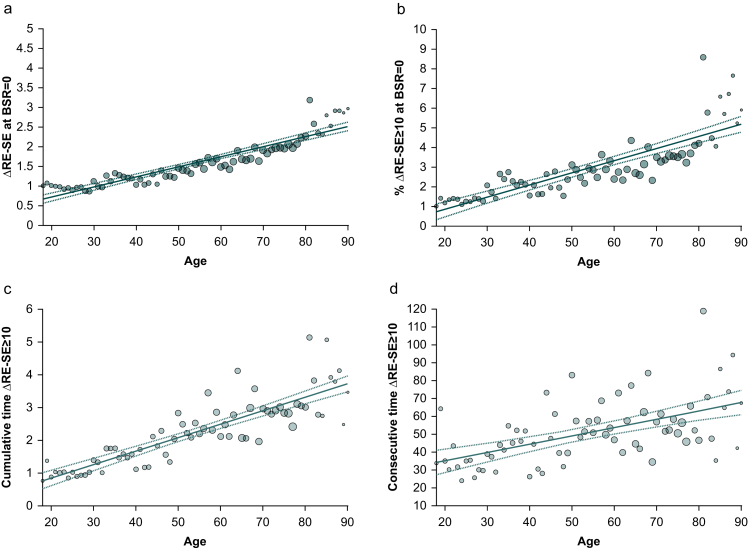
Differences between response entropy (RE) and state entropy (SE) (ΔRE-SE) during steady-state anaesthesia. (a) Mean ΔRE-SE at BSR=0 as a function of age. (b) Mean percentage of ΔRE-SE ≥10 within the 40–60 range. (c) Cumulative time (min) of ΔRE-SE ≥10 within the 40–60 range. (d) Consecutive time (s) of ΔRE-SE ≥10 within the 40–60 range. BSR, burst suppression ratio.

Not only did RE and SE values increase with age, but so did the ΔRE-SE. We confirmed this finding by calculating the mean difference during steady-state anaesthesia (Fig. 4a). There was a significant linear increase with advancing age (age *vs* mean ΔRE-SE: rho=0.96 [0.93–0.97], adj. *R*^2^=0.85, *P*<0.001), irrespective of the anaesthesia regime used (Supplementary Fig. S7). As an age-dependent increase of ΔRE-SE has already been demonstrated based on raw EEG,^19^ and the clinical relevance of mean ΔRE-SE between 0.5 and 3.5 might appear questionable, we focused on ΔRE-SE ≥10. We discovered a very strong positive correlation between the frequency of these occurrences and age (age *vs* % ΔRE-SE ≥10: rho=0.92 [0.85–0.95], adj. *R*^2^=0.70, *P*<0.001). This suggests that older patients experienced more ‘*arousal*’ during steady-state anaesthesia, as indicated by the monitor (Fig. 4b). Older patients spent significantly more cumulative and consecutive time in ΔRE-SE ≥10 constellations during steady-state anaesthesia (Fig. 4c and d). Being within the manufacturer-recommended SE index range did not prevent concurrent ΔRE-SE ≥10 in older patients.

## Discussion

In this retrospective analysis, we investigated the reliability of the GE Entropy™ module in helping to guide anaesthesia delivery to safe levels while reducing overly ‘*deep*’ or ‘*light*’ anaesthesia in a sizeable cohort of adult patients. Most SE values recorded during steady-state anaesthesia (BSR=0) fell below the manufacturer's recommended range. For volatile anaesthetics, we can ascertain adequate dosing and aaMAC values even increased with patient age. Older patients were more likely to exhibit contradictory index combinations, which include mean SE values within the recommended range despite BSR ≥5 or ΔRE-SE ≥10. These observations suggest clinically relevant limits to the algorithm's reliability as a ‘*one-size-fits-all*’ monitoring solution.

### Patient age and processed EEG indices

The influence of age on EEG characteristics during anaesthesia has been the subject of extensive investigation. Frontal EEG of older patients undergoing general anaesthesia exhibits faster oscillatory patterns with a more uniformly distributed spectral power across frequencies.^18^ Schultz and colleagues^29^ reported that applying a standardised propofol induction dose resulted in deeper EEG sedation stages in older patients but with less absolute power in the delta frequency band. These findings might explain higher SE values in older patients. In a study using entropy-guided TIVA with target-controlled infusions, young and old patients did not significantly differ regarding estimated propofol brain concentrations. Still, SE/RE values were significantly higher in the latter cohort.^30^ Compensating for intrinsic age effects constitutes a challenge for most commercial monitoring solutions. A study has already described the paradoxical positive correlation between patient age and higher BIS, even though older patients were exposed to higher aaMACs.^20^ A replay of intraoperative EEG to commercial monitoring systems revealed age-dependent increases in most indices, independent of anaesthetic concentrations.^19^ This contradicts the expected inverse relationship between anaesthetic concentrations and EEG indices. Our retrospective analysis confirms higher mean SE and RE during steady-state anaesthesia in older patients. Moreover, we found that the relationship between age and SE/RE appears to be nonlinear, with a third-degree polynomial model providing a significantly better fit than a linear model. This suggests a more complex interaction between age and EEG-based index values.

### Validity of manufacturer-recommended ranges for adequate anaesthesia

Most commercial monitors provide an index range for supposedly adequate anaesthesia, including the GE Entropy™ module. A major finding of this study is that patients spent most of their time during steady-state anaesthesia (BSR=0) below this corridor. In a prospective study, anaesthesiologists managed to maintain the target corridor for only 45% of SE values despite this constituting the primary intervention goal.^11^ Lehmann and colleagues^31^ further showed that adequate ‘*depth of anaesthesia*’ might depend on the monitor choice. When comparing SE levels with BIS 40 or 50, the SE showed no significant differences between the two levels but was in the range of 20–25. This is a remarkable discrepancy, as the same EEG is deemed ‘*adequate*’ by BIS but classified as ‘*too deep*’ by the GE Entropy™ module. In another study, BIS and SE indices showed only moderate agreement during TIVA, and the proportion of agreement decreased with patient age.^32^ It remains unclear how manufacturers defined the cut-off index values for ‘*adequate*’ anaesthesia and whether these values are linked to verifiable clinical endpoints. Our discovery that adherence to the recommended range does not prevent the occurrence of BSR ≥5 or a ΔRE-SE ≥10 seriously questions its clinical relevance, as neither overly ‘*deep*’ nor ‘*light*’ anaesthesia was prevented.

### Prevention of ‘*deep*’ anaesthesia through avoidance of burst suppression

Preventing overly ‘*deep*’ anaesthesia is one of the main goals of processed EEG indices. There is substantial evidence that, especially in older patients, burst suppression during general anaesthesia is linked to an increased incidence of postoperative delirium^33^ and even mortality.^34^^,^^35^ Therefore, it is advisable to avoid burst suppression during maintenance of anaesthesia. However, detection can be impaired with current (automated) BSR algorithms. As described in a case report, RE indicated prolonged wakefulness during general anaesthesia despite the underlying raw EEG displaying profound isoelectricity.^36^ The amplitude range of suppression episodes, specifically the low voltage or isoelectric EEG signal component of burst suppression (measured in μV), was significantly higher in undetected than in detected automated burst suppression cases.^37^ However, even if SE values adhere to the manufacturer-recommended range (SE 40–60), the occurrence of BSR ≥5 might not be preventable. Acceptable index values can be deceptive as the patient's EEG might already show burst suppression. Anaesthesia guidance based on indices requires caution by the user. It is advisable for anaesthesiologists to gain proficiency in interpreting the raw EEG signal, as recommended by experts and international guidelines.^2^^,^^38^ Additionally, a recent survey from Norway showed that 86% of anaesthesiologists and nurse anaesthetists desired further training in raw EEG interpretation.^39^

### Indicator of impending *‘arousal’* during anaesthesia: the difference between response entropy and state entropy

The difference between RE and SE should indicate an impending arousal reaction during general anaesthesia.^5^ This claim was confirmed in principle by a study evaluating RE/SE before and after tracheal intubation during general anaesthesia without neuromuscular blocking agents.^40^ Compared with other algorithms designed to detect arousal, ΔRE-SE was less sensitive in detecting painful stimulations but still showed a significant difference.^41^ One of the underlying mechanisms (besides facial EMG activation) might be beta arousal (higher power in the beta frequency range of 12–25 Hz), which can be observed after noxious stimuli.^42^ Neuromuscular blocking agents also significantly reduced ΔRE-SE.^16^ Our study reveals that baseline ΔRE-SE and ΔRE-SE ≥10 exhibit a significant age-dependent increase. The impact of age on ΔRE-SE reliability extends to steady-state anaesthesia. Even when anaesthesia is titrated to ‘*adequate*’ index ranges, ΔRE-SE ≥10 occurs. Anaesthesiologists should exercise caution when incorporating this parameter into clinical decision-making, especially in older patients. It is not yet well validated and is confounded by factors such as patient age and concentrations of anaesthetic, analgesic, and neuromuscular blocking agents.^16^^,^^17^

### Limitations

Our analysis did not include recordings of raw EEG traces and additional parameters, including drug doses for propofol and opioids, relaxometry, haemodynamic parameters, or type of surgical procedure. Consequently, we only evaluated volatile anaesthetic concentrations. Further, our findings regarding the age-dependent increase of ΔRE-SE ≥10 might be partially explained by lower doses of neuromuscular blocking agents in older patients and potential differences in the analgesic component of anaesthesia. Anaesthesia management followed no specified titration goal, but the manufacturer-recommended range was included in the monitor output and readily accessible. The BSR and RE/SE indices were logged simultaneously but based on different time windows, which could partly account for output constellations described in this study.

### Conclusions

Patient age significantly impacts the reliability of processed EEG indices including RE, SE, ΔRE-SE, and BSR. Most SE values persistently fell below the manufacturer-deemed adequate ‘*depth of anaesthesia*’ value range during steady-state anaesthesia conditions (BSR=0). Adherence to the ‘*adequate*’ index range did not prevent BSR, especially in older patients. On the other side of the spectrum, ΔRE-SE increased significantly with patient age, including excessive differences surpassing the proposed ‘*arousal*’ threshold even when within the ‘*adequate*’ index range. Relying solely on these indices poses a risk of misguiding anaesthesia, particularly in older patients. We advocate for age adjustments in processed EEG indices. This is crucial for future applications, including the integration of these indices into automated anaesthesia titration systems.^43^ Given the current limitations of processed EEG indices, anaesthesiologists should not only strive for proficiency in assessing the raw EEG, but also develop an understanding of its age-related changes.^39^ Interpreting EEG data in older patients during anaesthesia poses distinct challenges, as it is characterised by increased irregularities and differences in both burst suppression and non-burst suppression states.

## Authors' contributions

Contributed to the study's conception and design: all authors

Data curation: ME, MK, SS

Formal analysis, software: ME

Writing of the original draft: ME, MK, SS

Visualisation: ME

Conceptualisation: MK

Methodology: MK, SS

Project administration: MK

Writing and editing of original draft: MK, SK, GS

Supervision: MK, SS

Read and approved the final manuscript: all authors

## Declarations of interest

MK is named an inventor for a patent dealing with spectral EEG features and age (US Provisional Patent Application No. 62/914,183). GS and MK are named inventors for a patent filed on a novel method for intraoperative EEG monitoring (US Patent Application Serial No. 62/960,947). GS, MK, and SK are also named inventors of a patent dealing with EEG features during the emergence of anaesthesia (US Provisional Patent Application No. 63/459,294). MK received funding from Masimo Corporation, Narcotrend-Gruppe, Medtronic GmbH, and Fresenius Kabi Deutschland GmbH for conducting EEG-based training for anaesthesiologists. SS and ME declare that they have no conflicts of interest.
