# Supplementary material for: Impact of age on the reliability of GE Entropy™ module indices for guidance of maintenance of anaesthesia in adult patients: a single-centre retrospective analysis
Source: Br J Anaesth. 2025 Feb 6;134(4):1077–87. doi: 10.1016/j.bja.2024.11.050 (PMC11947563; doi:10.1016/j.bja.2024.11.050)
Supplement: Multimedia component 1 [file mmc1.pdf]

Online supplementary material for: *"Impact of Age on the Reliability of GE Entropy™ Module Indices in Anaesthesia Guidance for Adult Patients: A Retrospective Analysis"*

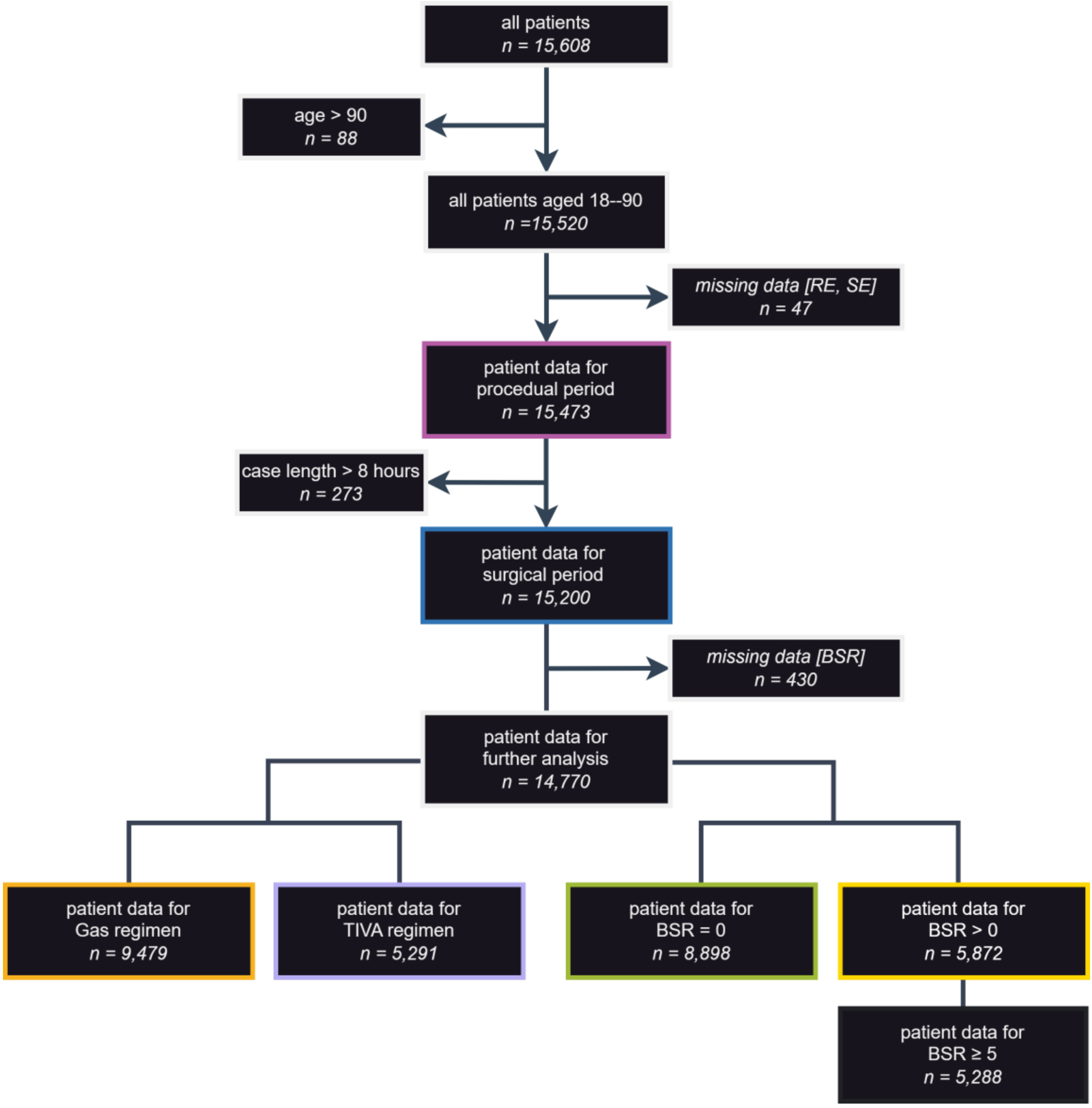

Fig. S1: Flowchart with selection process for different time periods and exclusion criteria.

Frame colours align with colours chosen to illustrate time periods in subsequent plots.

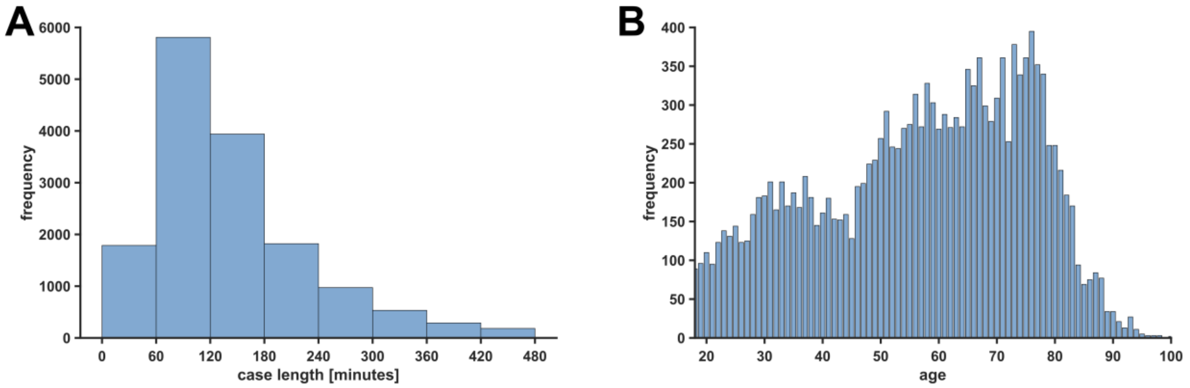

Fig. S2: Frequency distribution of surgery duration and patient age

A) Histogram of surgery duration of all patients included.

B) Histogram of the age distribution of all patients included.

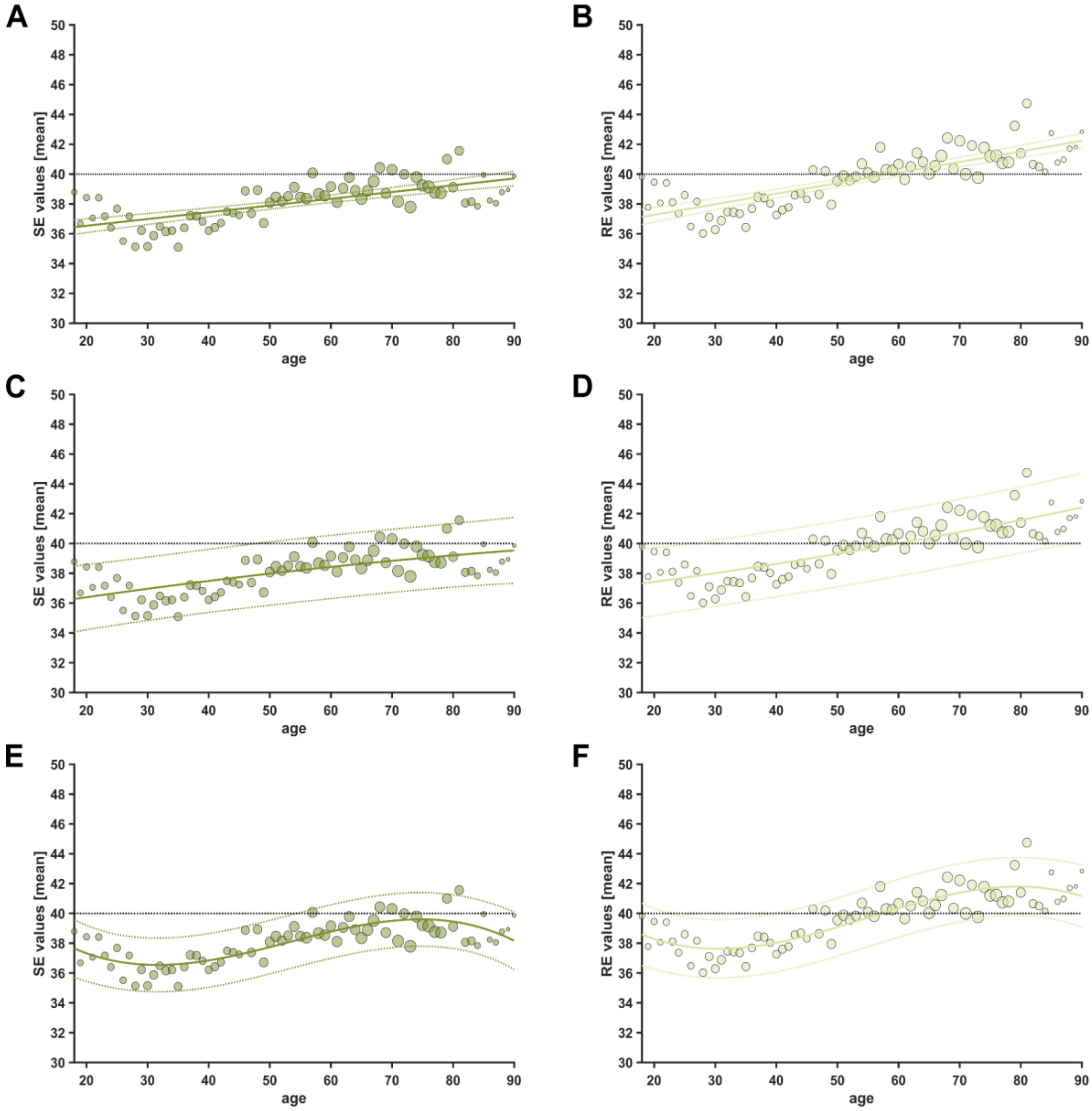

Fig. S3: Association of patient age and State Entropy (SE) and Response Entropy (RE) for the steady-state anaesthesia period using linear, second-degree polynomial, and third-degree polynomial regression models.

The lower threshold for adequate anaesthesia, as recommended by the manufacturer (40), is indicated with a dotted line. Dot sizes per age are scaled according to group size.

A) Patient age and mean SE with a linear fit.

B) Patient age and mean RE with a linear fit.

C) Patient age and mean SE with a second-degree polynomial fit.

D) Patient age and mean RE with a second-degree polynomial fit.

E) Patient age and mean SE with a third-degree polynomial fit.

F) Patient age and mean RE with a third-degree polynomial fit.

Supplementary Table 1: Results for likelihood ratio test, comparing a linear model with second-degree and third-degree polynomial models for the association between SE and patient age (steady-state anaesthesia). Adj.: adjusted, AIC: Akaike information criterion, BIC: Bayesian information criterion, RSS: residual sum of squares.

model	Adj. R2	AIC	BIC	RSS	P
**linear**	0.45	215.38	219.96	77.34	-
**quadratic**	0.45	216.95	223.82	76.89	0.52
**cubic**	0.61	193.01	202.17	53.89	<0.001

Supplementary Table 2: Results for likelihood ratio test, comparing a linear model with second-degree and third-degree polynomial models for the association between RE and patient age (steady-state anaesthesia). Adj.: adjusted, AIC: Akaike information criterion. BIC: Bayesian information criterion. RSS: residual sum of squares.

model	Adj. R2	AIC	BIC	RSS	P
**linear**	0.66	221.23	225.81	83.79	-
**quadratic**	0.65	222.75	229.62	83.24	0.50
**cubic**	0.73	204.92	214.09	63.46	<0.001

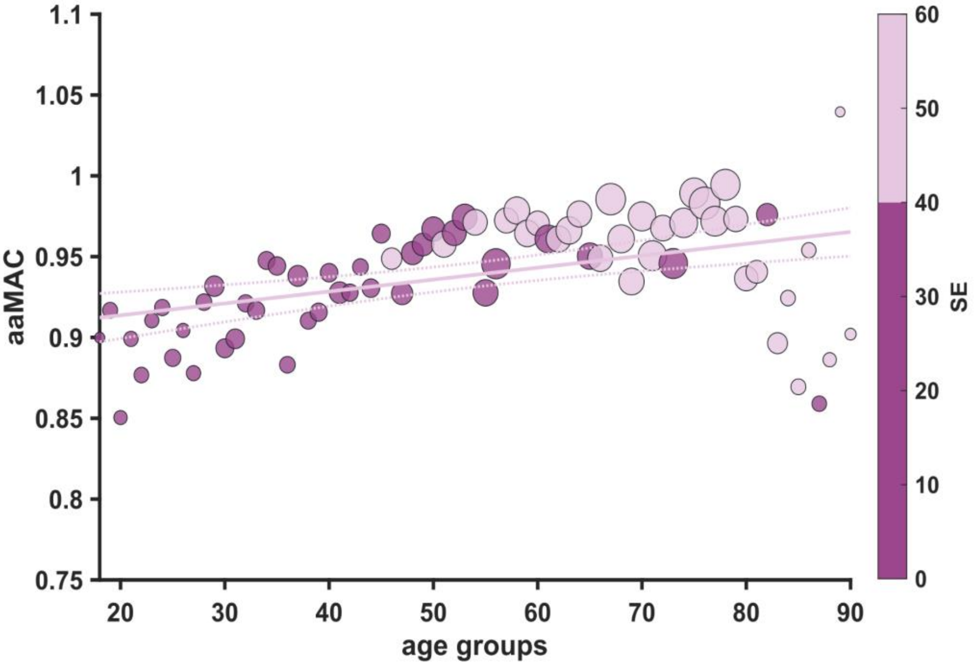

Fig. S4: Mean age-adjusted minimal alveolar concentration (aaMAC) of Sevoflurane over age groups with corresponding mean State Entropy (SE) values during steady-state anaesthesia.

Mean SE values below the threshold for adequate anaesthesia (<40) are coloured with dark pink. Adequate SE values (≥40) are shown in light pink. Dot sizes per age are scaled according to group size*. Linear model: 0.90*age + 0.0007 [0.0004, 0.001], age versus aaMAC: rho=0.47 [0.20–0.71], adj. $R2$=0.18, P<0.001.*

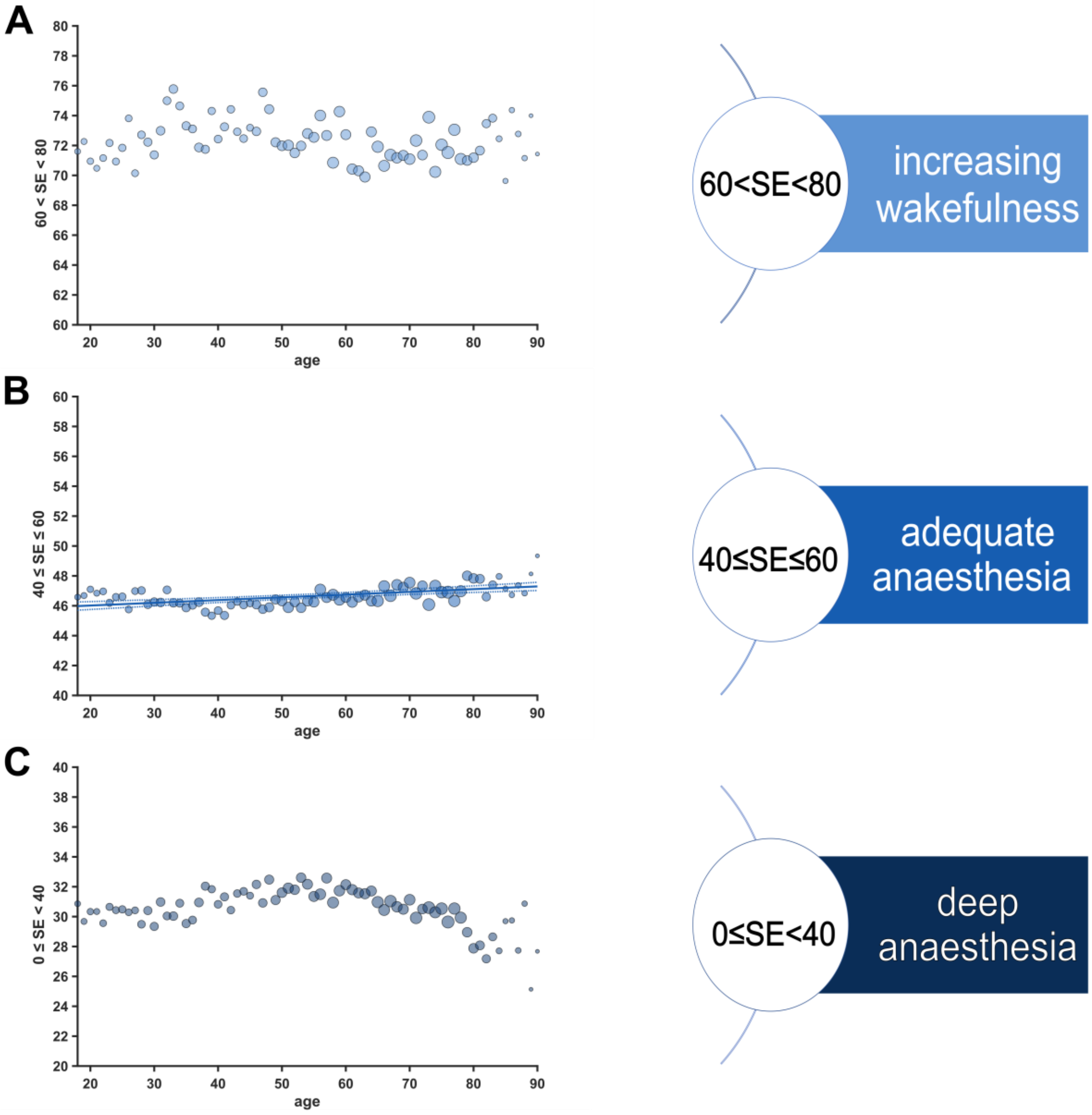

Fig. S5: Mean State Entropy (SE) value distribution during the steady-state anaesthesia period in relation to the manufacturer-recommended range

Mean SE values within the recommended range (40–60) during steady-state anaesthesia demonstrated a significant, linear upward trend. Dot sizes per age are scaled according to group sizes. Due to the poor quality of linear regression fits for mean values above or below the range, they were not included for A and C.

A) Mean SE values between 60–80 during steady-state anaesthesia.

B) Mean SE values between 40–60 during steady-state anaesthesia. Linear regression fit in the corresponding colour with confidence interval as dotted lines.

C) Mean SE values below 40 during steady-state anaesthesia.

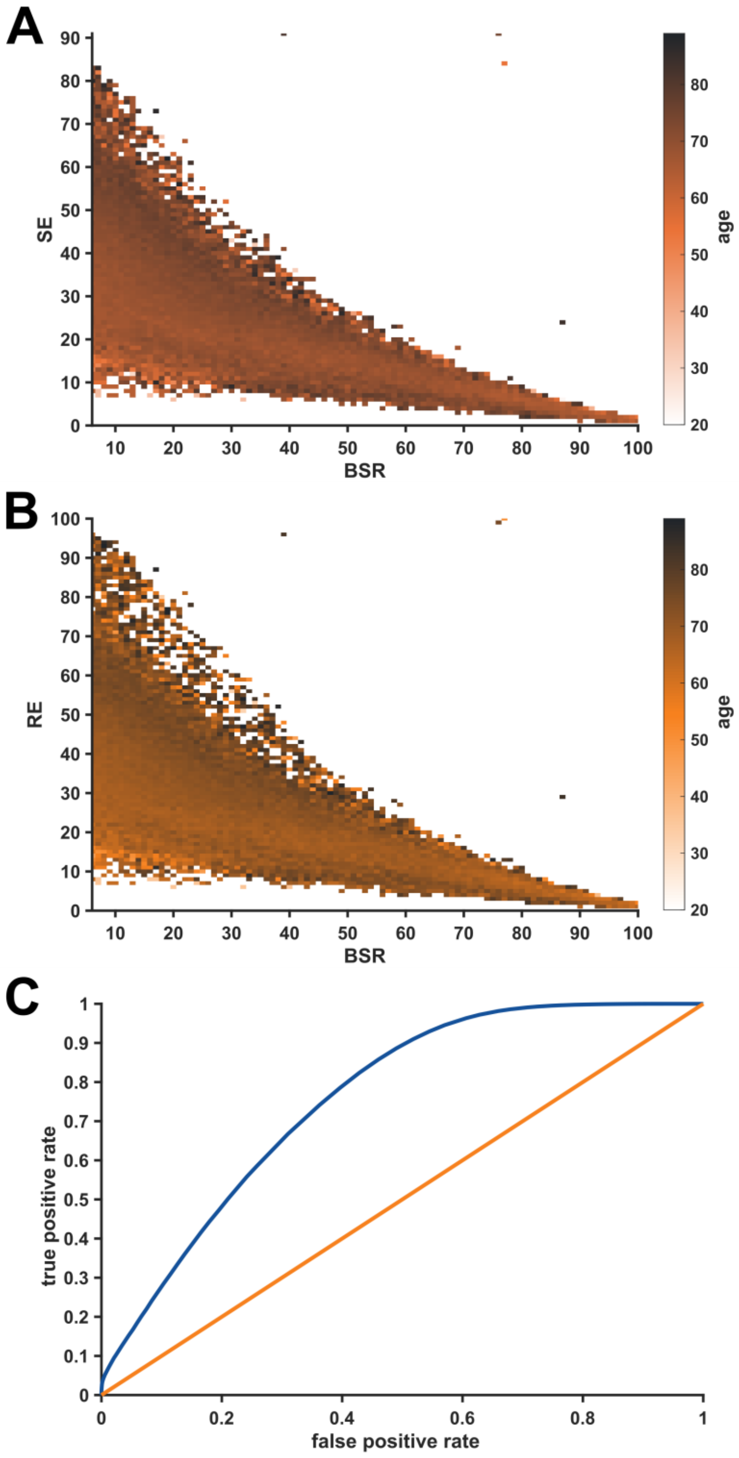

Fig. S6: Heat maps of State Entropy (SE) and Response Entropy (RE) index values as a function of burst suppression ratio (BSR) and patient age.

A) SE over BSR during the surgical period. The colour bar indicates the patient's age.

B) RE over BSR during the surgical period. The colour bar indicates the patient's age.

C) Receiver operating characteristic curve evaluating the performance of SE as a classifier to discriminate between burst suppression occurrence or absence. The orange diagonal line illustrates a random classifier (AUROC 0.5) for comparison.

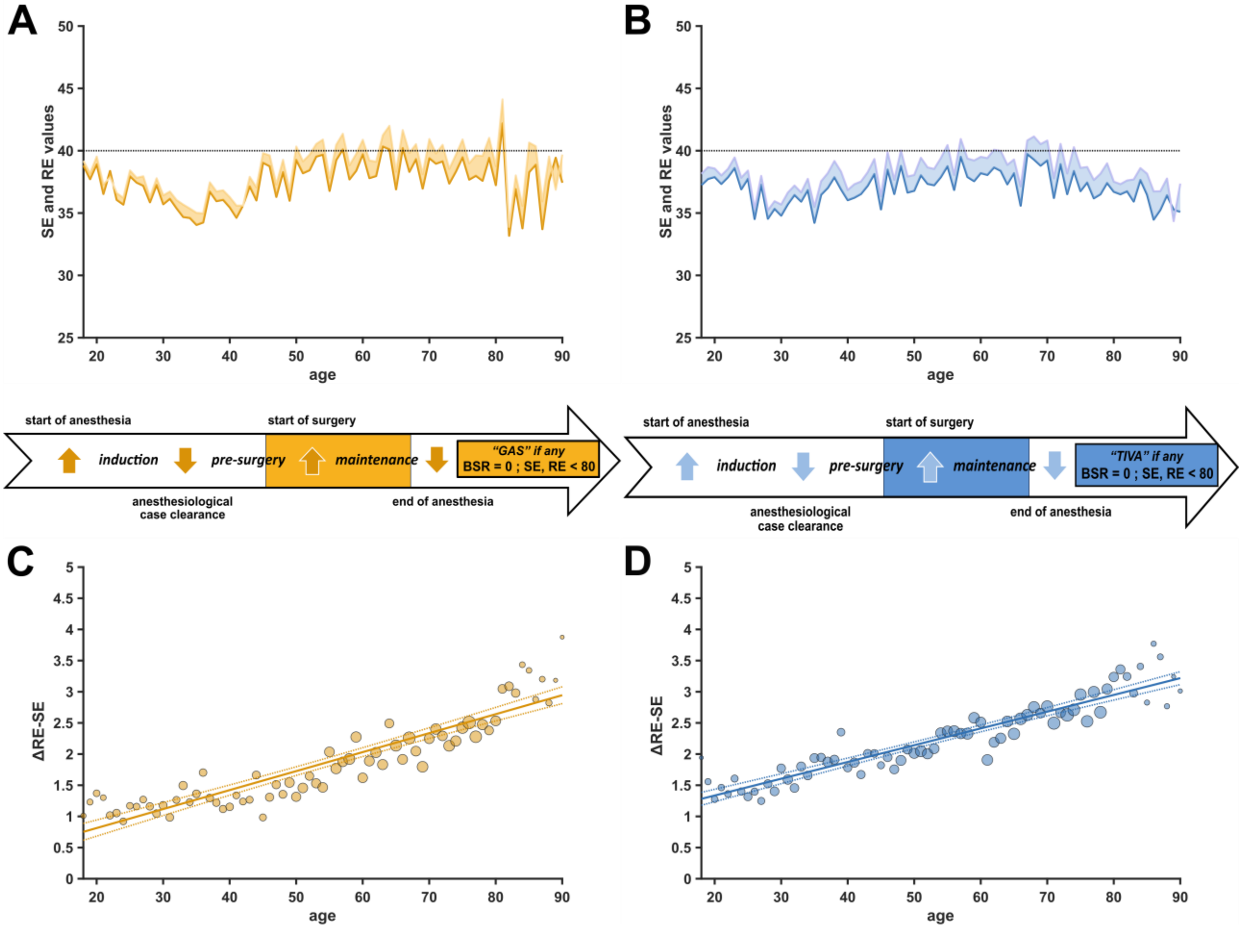

Fig. S7: Mean values for State Entropy (SE) and Response Entropy (RE) during steady-state anaesthesia for maintenance by volatile anaesthetics and total intravenous anaesthesia (TIVA).

The lower threshold of the manufacturer-provided range for adequate anaesthesia (40) is highlighted with a dotted line. The age-dependent increase of the RE-SE difference (Δ) is present in both regimes.

A) Mean SE (dark orange) and RE (light orange) for the volatile anaesthetic gas regimen during steady-state anaesthesia. Below, a timeline delineates the selected period.

B) Mean SE (dark blue) and RE (light blue) for the TIVA regimen during steady-state anaesthesia.

C) ΔRE-SE difference for the volatile anaesthetic gas regimen. Linear regression fit in the corresponding colour and the confidence interval as dotted lines.

D) ΔRE-SE difference for the TIVA regimen.
